# Epigenetic Clocks as Biomarkers for Bone Aging: Evidence From a Twin Study

**DOI:** 10.1111/acel.70204

**Published:** 2025-09-14

**Authors:** Mette Soerensen, Florence Figeac, Kaare Christensen, Moustapha Kassem

**Affiliations:** ^1^ The Danish Twin Registry and the Research Unit for Epidemiology, Biostatistics, and Biodemography, Department of Public Health University of Southern Denmark Odense Denmark; ^2^ Department of Clinical Genetics Odense University Hospital Odense Denmark; ^3^ Molecular Endocrinology Department, Department of Endocrinology Odense University Hospital Odense Denmark; ^4^ Department of Cellular and Molecular Medicine, Faculty of Health, and Medical Sciences University of Copenhagen Copenhagen Denmark; ^5^ College of Medicine and Health Sciences and Center for Biotechnology Khalifa University Abu Dhabi UAE

**Keywords:** age acceleration, bone aging, bone fracture, bone mineral density, bone turn over markers, epigenetic clocks, osteoporosis, twins

## Abstract

Osteoporosis is driven by skeletal aging and increases the risk of fragility fractures. Skeletal aging is influenced by epigenetic mechanisms; however, the link to the epigenetic clocks, that is, the suggested biomarkers of biological aging intensively studied within aging research, remains largely unexplored. We investigated the association of eight epigenetic clocks—Horvath, IEAA, Hannum, EEAA, PhenoAge, GrimAge, DunedinPoAm, DunedinPACE—and methylation‐based telomere length (mTL) measured at intake (1997, 2007, or 2008–11) with register‐based incident fracture and osteoporosis diagnoses in 310 (Discovery cohort), and 777 (Replication cohort) twins, derived from nationwide population‐based surveys (end‐of‐follow‐up: 2022). For the 288 youngest twins, bone mineral density (BMD), and bone turnover markers C‐terminal telopeptide (CTX) and amino pro‐collagen type 1 N‐terminal propeptide (P1NP) were available. Analyses were conducted at both the individual and twin‐pair levels, reducing genetic and environmental confounding. A consistent pattern emerged: increased epigenetic age, faster pace of aging, and shorter mTL associated with a higher risk of fractures, or osteoporosis. Notably, DunedinPoAm, DuninPACE, and GrimAge demonstrated the most robust associations. DuninPACE displayed the largest effects; hazard ratios ranging from 1.29 to 3.17, reflecting a 29%–217% increased hazard for diagnosis pr. one standard deviation increase in DuninPACE. The same directions of effects were seen for decreasing CTX and P1NP levels, suggesting bone remodeling impairment for individuals with higher biological age. Lastly, sex‐ and age‐specific analysis of BMD indicated the same direction of effect for DunedinPoAm, and GrimAge in older females. These findings suggest that epigenetic clocks may serve as biomarkers for bone aging.

## Graphical abstract

We investigated whether individuals with osteoporosis or fragility fracture are biologically older, as determined by the epigenetic clocks (biomarkers of aging). As the first of its kind, the study suggests that individuals with osteoporosis or fragility fractures exhibit accelerated aging based on their biological age determined by the epigenetic clocks.

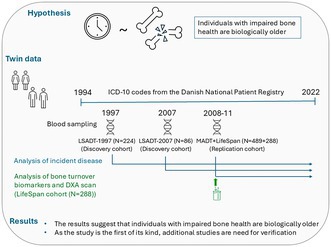

## 1 Introduction

Osteoporosis is a common disease affecting the aging population; 29% of all women and 14% of all men develop osteoporosis during their lifetime (Clynes et al. 2020). Osteoporosis is characterized by deterioration of bone tissue microarchitecture leading to bone fragility and an increased risk of bone fracture, commonly in the hip and spine, following minimal trauma (Compston et al. 2019). For the individual, such a fracture leads to loss of mobility, independence, a decreased quality of life, and an increased risk of mortality (Rachner et al. 2011). Hence, osteoporosis and age‐related bone fractures pose a major health care problem, especially considering the worldwide increase in numbers of older individuals.

Traditionally, osteoporosis has been considered to be caused by age‐related changes in sex steroids; yet recent research points to accelerated skeletal aging taking off before, and independently of, changes in hormone levels (Manolagas 2010). The accelerated aging is caused by, among others, age‐related impairment of osteoblastic function (Kassem and Marie 2011) or changes in the bone micro‐environment affecting osteoblastic function (Abdallah et al. 2006; Stenderup et al. 2003; Andreasen et al. 2018). Also, osteoporosis is associated with other age‐related diseases, for example, osteopenia, neurodegenerative, and cardiovascular diseases (Clynes et al. 2020), and as such, osteoporotic individuals exhibit an accelerated aging phenotype.

Only a few studies have studied the link between osteoporosis or fracture risk with biological age as estimated using the epigenetic clocks, that is, the suggested biomarkers of biological aging intensively studied within aging research during the last decade (Margiotti et al. 2023). These clocks are based on genome‐wide DNA methylation array data and are divided into first‐generation clocks, like the Horvath and Hannum clocks, generated using chronological age as the outcome, and second‐generation and third‐generation clocks, like the PhenoAge, DunedinPoAm, DunedinPACE, GrimAge, and mTL clocks, generated using age‐related biochemical measurements, mortality, or leukocyte telomere length (LTL) as outcomes (Margiotti et al. 2023). Hence, these clocks are generated in different ways; yet, they are all considered to capture biological aging per se. For the clocks measured in years, the difference between the estimated biological age (i.e., the clock estimate) and the chronological age, termed AgeAcceleration, is employed as a measure of higher and lower biological age, termed accelerated, and deaccelerate aging, respectively. The same goes for the increase, respectively decrease, in the DunedinPoAm and DunedinPACE pace of aging.

The contribution of epigenetics to age‐related changes in bone tissue is evident (Fuggle et al. 2022), for example, age‐related changes in DNA methylation predict impaired osteoblastic functions (Zhong et al. 2024). In a small sample comparing 32 osteoporosis patients versus 16 controls, Fernandez‐Rebollo et al. (2018) did not detect any significant differences in DNA methylation age, employing the Horvath, Hannum, and Weidner clocks. Also, two studies have investigated the association with bone mineral density (BMD): Simpkin et al. (2017) investigated the Horvath clock in 1018 children of the ALSPAC cohort (age 7–19), and Shiau et al. (2024) investigated 118 cases and 72 controls from the Women's Interagency HIV Study, employing six clocks (the Horvath, PhenoAge, GrimAge, and mTL clocks, and the intrinsic epigenetic age acceleration (IEAA), and extrinsic epigenetic age acceleration (EEAA) clocks, derived from the Horvath, and Hannum clocks, respectively). Both studies did not detect a statistically significant association. On the other hand, del Real et al. (2017) investigated the Horvath clock in bone marrow stromal stem cells established from femoral heads obtained during hip replacement surgery of 22 osteoporotic patients with femoral head fracture and 17 patients with osteoarthritis, and found accelerated epigenetic aging for the osteoporotic patients.

One challenge when investigating the association between a molecular marker and an age‐related phenotype is that such association is confounded by genetic variation. To overcome this, twin pairs, where one twin develops the trait of interest while the co‐twin does not, for example, one twin experiences a bone fracture while the co‐twin does not, can be investigated. This design is known as the discordant twin pair design, and it is statistically very powerful (Li et al. 2018). This design also corrects for confounding environmental factors, especially related to the shared early life environment.

**FIGURE 1:**
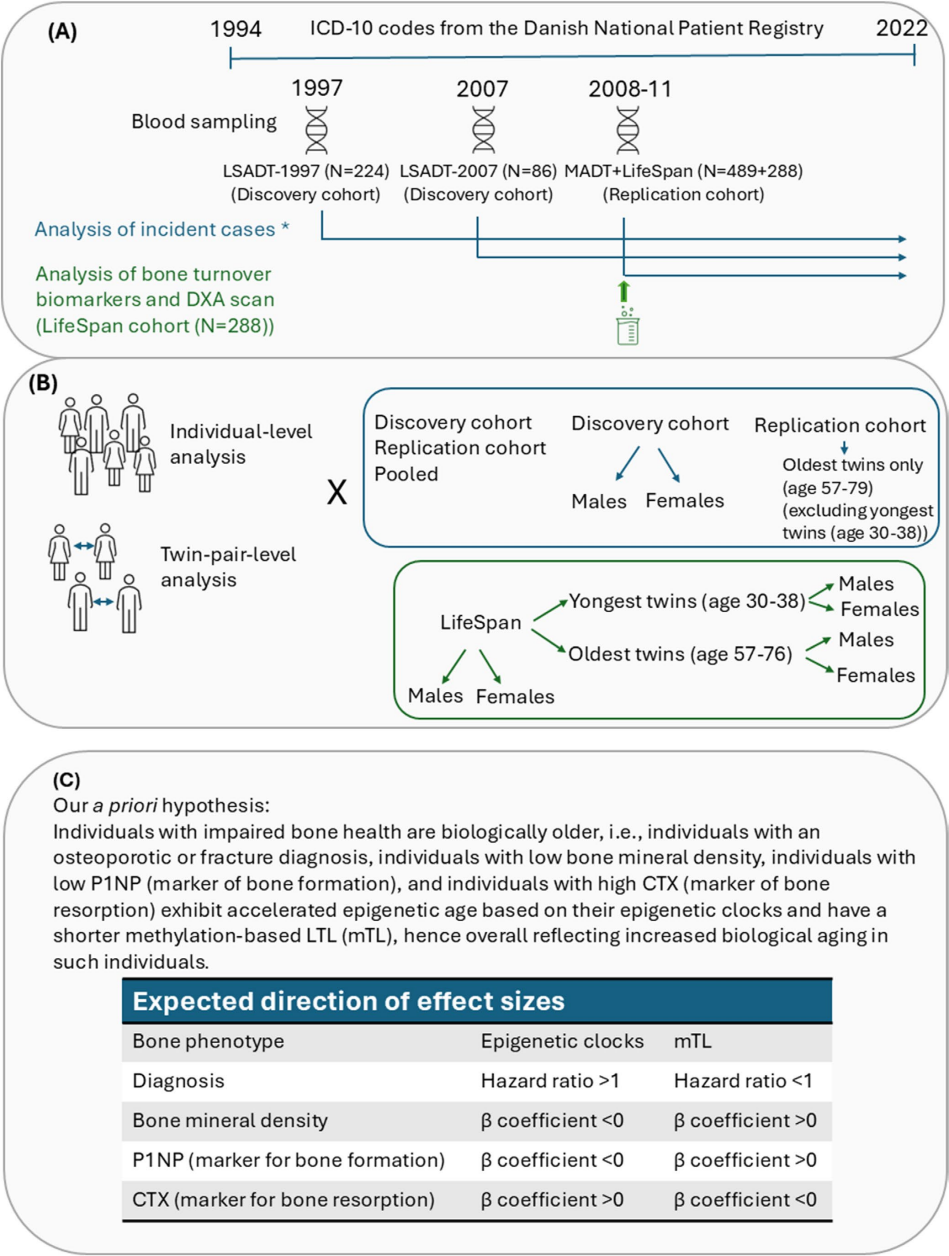
Study pipeline. (A) Timeline of the register diagnose data used for analysis of incident fragility fracture and osteoporosis diagnoses (blue) and the data on DXA scans and bone turnover biomarkers analyzed in the LifeSpan cohort (green). (B) Statistical analyses performed for diagnose data (blue) and DXA scans and biomarker data (green). All analyses were performed both at the individual level and at the twin pair (intra‐pair) level. (C) Our a priori hypothesis and expected directions of effect. CTX, C‐terminal telopeptide; LTL, leukocyte telomere length; P1NP, amino pro‐collagen type 1 N‐terminal propeptide (P1NP). *Analysis of time to first diagnosis, death or end‐of‐follow‐up. Cases were individuals with diagnosis after blood sampling, yet no diagnosis before blood sampling. Individuals with analysis before blood sampling were excluded from analysis.

Here, we investigated the association between nine epigenetic clocks and incident diagnoses of fractures or osteoporosis obtained from the Danish National Patient Registry (DNPR) for a nationwide population‐based sample of 1087 adults and older twins, including a Discovery cohort (*N* = 310) and a Replication cohort (*N* = 777). As LTL is also a suggested biomarker of biological aging, the mTL clock was among the nine clocks. A description of the clocks investigated is given in File S1. In a subset of the sample (*N* = 288) data on BMD and the bone turnover markers C‐terminal telopeptide (CTX) and amino pro‐collagen type 1 N‐terminal propeptide (P1NP) were also available. Our a priori hypothesis was that individuals with osteoporosis or fracture diagnosis, individuals with low BMD, individuals with low P1NP (marker of bone formation), and individuals with high CTX (marker of bone resorption) exhibit accelerated epigenetic age based on their epigenetic clocks and have a shorter methylation‐based LTL, hence overall reflecting increased biological aging in such individuals. The study pipeline is displayed in Figure 1.

## 2 Materials and Methods

A detailed description of the materials and methods can be found in the Data S1; while the study pipeline is illustrated in Figure 1.

### 2.1 Study Population

The descriptives of the study population can be seen in Table 1: 1087 twins were drawn from three population‐based, nationwide surveys from the Danish Twin Registry (Pedersen et al. 2019): The Longitudinal Study of Aging Danish Twins (LSADT), The Middle Age Danish Twin study (MADT), and the Birthweight‐Discordant Study (hereon called LifeSpan): the LSADT twins composed the Discovery cohort (*N* = 310), while the MADT and LifeSpan twins composed the Replication cohort (*N* = 777). For all cohorts, comprehensive interview‐based questionnaires, examinations, and blood sampling had been conducted. Informed consent was obtained from all participants, and the surveys were approved by the Regional Scientific Ethical Committees for Southern Denmark (S‐VF‐19980072, S‐VF‐20040241, and S‐20090033) and conducted in accordance with the Helsinki II declaration.

**TABLE 1:** Characteristics of the study population.

			Discovery cohort	Replication cohort
No. individuals			310	777
No. twin pairs (no. monozygotic pairs: dizygotic pairs)			155 (119:36)	388 (388:0)
*Survey data*				
Sex [females (%): males (%)]			214 (69.0):96 (31.0)	363 (46.7):414 (53.3)
Age at blood sampling, years [mean (SD), range]			81.2 (4.66), 73–91	60.1 (13.43), 30–79
Height at blood sampling, cm [mean (SD), range]			162.9 (8.71), 140–183	169.9 (9.32), 147–205
Weight at blood sampling, kg [mean (SD), range]			64.7 (12.52), 39–105	76.1 (14.48), 35–145
Current smoker at blood sampling [yes (%)/no (%)]			67 (21.6)/243 (78.4)	172 (22.1)/605 (77.9)
Alcohol 3 units per day at blood sampling [≥ 3 units (%)/< 3 units (%)]			19 (6.1)/291 (93.9)	77 (9.9)/700 (90.1)
Charlson co‐morbidity Index at blood sampling [0 (%), 1 (%), 2 (%), 3–8 (%)]			184 (59.4), 64 (20.6), 40 (12.9) 22 (7.1)	585 (75.3), 89 (11.4), 73 (9.4), 30 (3.9)
*Diagnoses*				
Fragility fractures	Before blood sampling	No. individuals (%)	42 (13.5)	60 (7.7)
	After blood sampling	No. individuals (%)	102 (32.9)	79 (10.2)
		No. twin pairs with diagnosis [discordant twin pairs: concordant twin pairs (%)]^a^	56 (36.1):23 (14.8)	69 (17.8):5 (1.3)
Osteoporosis	Before blood sampling	No. individuals (%)	15 (4.8)	32 (4.1)
	After blood sampling	No. individuals (%)	28 (9.0)	92 (11.8)
		No. twin pairs with diagnosis [discordant twin pairs: concordant twin pairs (%)]^a^	22 (14.2):3 (1.9)	66 (17.0):13 (3.4)
*Epigenetic clocks*				
Chronological age at blood sampling [mean (SD)]			81.2 (4.66)	60.1 (13.43)
*PC Horvath clock*				
DNAmAge [mean (SD)]			68.85 (6.70)	53.49 (11.02)
Age Acceleration Residual [mean (SD)]			−5.03 × 10^−11^ (6.34)	9.52 × 10^−10^ (4.53)
IEAA [mean (SD)]			3.20 × 10^−9^ (4.63)	7.44 × 10^−10^ (3.83)
*PC Hannum clock*				
DNAmAge [mean (SD)]			78.92 (6.75)	62.81 (11.50)
AgeAccelerationResidual [mean (SD)]			9.55 × 10^−9^ (6.28)	2.74 × 10^−9^ (4.56)
*BioAge4HAStatic clock*				
DNAmAge [mean (SD)]			84.89 (8.13)	64.51 (15.61)
EEAA [mean (SD)]			−7.73 × 10^−9^ (7.32)	7.89 × 10^−10^ (5.72)
*PC PhenoAge clock*				
DNAmAge [mean (SD)]			74.88 (7.69)	56.13 (12.57)
AgeAccelerationResidual (mean (SD))			−4.46 × 10^−9^ (6.67)	−3.97 × 10^−9^ (5.37)
*PC GrimAge clock*				
DNAmAge (mean (SD))			87.67 (4.79)	71.74 (11.54)
AgeAccelerationResidual [mean (SD)]			3.56 × 10^−9^ (3.45)	8.09 × 10^−9^ (3.94)
*DunedinPoAm clock*				
DNAmAge [mean (SD)]			0.92 (0.08)	0.92 (0.09)
*DunedinPACE clock*				
DNAmAge [mean (SD)]			1.14 (0.13)	1.06 (0.13)
*PC mTL clock*				
DNAmAge [mean (SD)]			6.61 (0.20)	6.93 (0.27)
**Bone phenotypes in the LifeSpan Cohort**				
*DXA*				
Hip total [*N*, mean (SD), range]			272, 0.98 (0.15), 0.45–1.44	
Femoral neck [*N*, mean (SD), range]			272, 0.87 (0.18), 0.34–1.62	
Lumbar spine [*N*, mean (SD), range]			264, 1.03 (0.16), 0.64–1.72	
*Bone biomarkers*				
CTX [*N*, mean (SD), range]			282, 0.44 (0.20), 0.07–1.11	
P1NP [*N*, mean (SD), range]			282, 49.76 (20.29), 12.22–140.3	

Abbreviations: AgeAccelerationResidual, residuals obtained from a regression analysis of DNAmAge (outcome) and chronological age (exposure); DNAmAge, DNA methylation age; DXA, dual‐energy X‐ray absorptiometry; mTL, estimate of telomere length based on DNA methylation data; No., number of individuals; SD, standard deviation.

^a^ For the incident cases, the number of discordant twin pairs and concordant twin pairs are listed; the discordant twin pairs are pairs where only one twin obtains a diagnosis, while the co‐twin does not. The concordant twin pairs are twin pairs where both receive a diagnosis, yet at different time points; hence, the concordant twin pairs also contribute to the analysis of incident cases (stratified cox regression analysis).

International Classification of Diseases (ICD) 10 codes were available from the DNPR (see https://sundhedsdatastyrelsen.dk) until the 23rd of November 2022; two disease groups were investigated: fragility/osteoporotic fractures (hereon called fragility fractures) and osteoporosis (File S2 for details). As the focus was on incident disease, only individuals holding a minimum one diagnosis after blood sampling (blood sampling took place in 1997 or 2007 (LSADT) and 2008–2011 (MADT/LifeSpan)) yet no diagnoses before blood sampling (diagnose coverage back to 1994), within the disease group of interest, were included. Similarly, within each disease group, the twin pairs discordant for incident diagnose status were found, that is, the twin pairs where one co‐twin received a diagnosis while the co‐twin remained disease free, and twin pairs where both twins received a diagnosis yet at different time points. To investigate time to diagnosis after blood sampling, mortality status was obtained from the Danish Central Person Register (see https://sundhedsdatastyrelsen.dk), assessed also on the 23rd of November 2022; 304 out of the 310 individuals of the Discovery cohort and 125 out of the 777 individuals of the Replication cohort had died.

Finally, the LifeSpan cohort (see File S3 for descriptives) also underwent whole body dual‐energy X‐ray absorptiometry (DXA) scanning and analyses for several biochemical markers (e.g., Frost et al. 2013). In the present study DXA measurements of BMD of the total hip, femoral neck, and lumbar spine (g/m^2^) were analyzed, as well as serum measurements of bone turnover markers: CTX and P1NP (μg/L).

### 2.2 The Epigenetic Clocks

We calculated six different measures of epigenetic age measured in the units of years: Horvath (Horvath 2013), Hannum (Hannum et al. 2013), PhenoAge (Levine et al. 2018), GrimAge (Lu, Quach, et al. 2019), IEAA, and EEAA (Chen et al. 2016), two measuring the pace of biological aging [DunedinPoAm (Belsky et al. 2020), DunedinPACE (Belsky et al. 2022)], and one measuring LTL [mTL (Lu, Seeboth, et al. 2019)]. The Horvath, Hannum, PhenoAge, GrimAge, and mTL were computed as principal component (PC) clocks using the method published by Higgins‐Chen et al. (Higgins‐Chen et al. 2022), while DunedinPoAm38 and DunedinPACE were obtained as described in the original publications (Belsky et al. 2020, 2022), and EEAA was acquired using scripts kindly provided by Drs. Steve Horvath and Ake Lu (University of California). To ensure statistical analyses in a consistent manner, we defined epigenetic age acceleration (AgeAccel) as the residuals obtained from regressing the estimators on chronological age for the clock measured in units of years. As seen in File S4, as expected these epigenetic clocks in general correlated to the chronological age, while the AgeAccels did not. Before statistical analysis, all clocks were standardized to a mean of 0 and a standard deviation of 1. This was done separately for the Discovery and the Replication cohorts for the analyses of diagnoses, and separately for the LifeSpan cohort for the analyses of BMD and bone turnover markers.

### 2.3 Statistical Analyses

Analyses were performed at the individual level, and at the twin pair level; the latter with the purpose of investigating intra‐pair differences, hence adjusting for environmental and genetic confounders. All analyses were performed using STATA17 (Stata Corporation, College Station, TX, USA), and *p* values were two‐sided. We defined statistical significance as *p* < 0.05.

At the individual level, Cox proportional hazards regression with age as the timescale (delayed entry at blood collection) was performed, adjusting for sex by using sex‐specific baseline hazards. To account for dependency between twins in each pair, we used the robust estimator of variance (cluster function in STATA). Furthermore, based on the FRAX score evaluating the risk of fracture (De Laet et al. 2005) we performed two additional analyses: (1) in addition to age and sex, adjusting for current smoking status (yes/no) and drinking habits (< or ≥ 3 units per day); and (2) further adjusting for height (cm), weight (kg), and the Charlson co‐morbidity index (Christensen et al. 2011). These additional adjustments overall led to the same conclusions; the results are listed in the Data S1. For analysis at the twin pair level, a stratified Cox regression model (stratifying by twin pair ID) was used, adjusting for the same co‐variates as described above.

For analysis of bone phenotypes in the LifeSpan cohort, linear regression was performed with the epigenetic clocks as outcomes, adjusting for the same covariates as above. To account for dependency between twins in a pair, we used the Hubert‐White‐Sandwich (robust) estimator of variance (cluster function in STATA). In the twin pair level analysis, intrapair differences were investigated by fitting a fixed‐effects model with a within twin pair regression estimator (xtreg command with fe option in STATA).

All results of the present study are listed as tables, as well as forest plots, in the Files S5–S20. Forest plots were generated in R (version 4.4.1) using the ggplots2 library.

## 3 Results

### 3.1 Association Analyses of Bone Diagnoses and Biological Aging

#### 3.1.1 Individual Level Analysis

**FIGURE 2:**
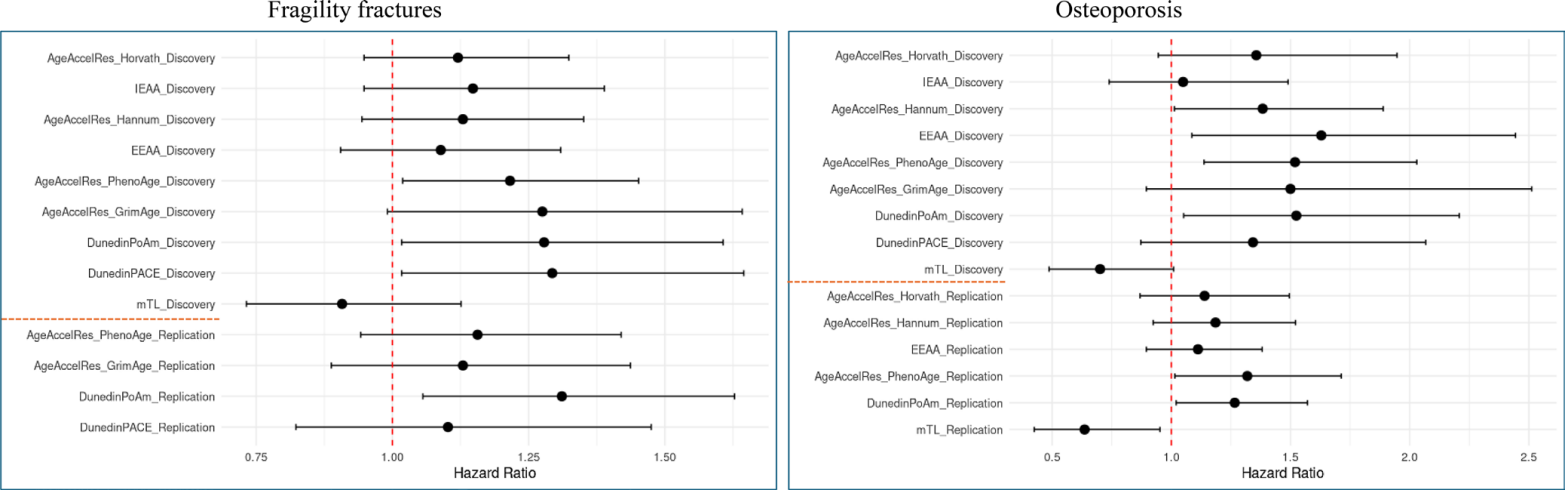
Association analysis at the individual level of fragility fracture and osteoporosis diagnoses after blood sampling. The red dotted line indicates a hazard ratio of 1, while the orange dotted line separates the Discovery cohort and the Replication cohort. For the Replication cohort, the epigenetic clocks with *p* values < 0.1 in the Discovery Cohort were aimed replicated.

As seen in Figure 2 and File S5, the Discovery cohort revealed several significant or borderline significant associations to fragility fractures or osteoporosis, with DunedinPoAm and PhenoAge being significant in both diagnostic groups. Of all clocks, DunedinPACE displayed the largest effect size for fragility fractures (HR = 1.29), while EEAA showed the largest effect size for osteoporosis (HR = 1.63) (File S5). In the replication sample (Figure 2, bottom), the same directions of effects were seen, with significant replication of the DunedinPoAm in both disease groups and of PhenoAge and mTL for osteoporosis.

The consistent pattern in effect sizes seen in the Discovery cohort indicates a decreased risk of obtaining a diagnosis per unit increase in standardized age‐adjusted LTL (mTL), while for the remaining clocks it indicates an increased risk of obtaining a diagnosis per unit increase in standardized epigenetic age; that is, these directions of effect are in correspondence with our a priori hypothesis of increased biological age in individuals with impaired bone health.

In the Replication cohort, a similar pattern was observed across clocks (File S5), and analyzing both cohorts together did for the most part move the associations away from the null compared to the Discovery cohort (File S5); for osteoporosis, this was seen for all the second and third generation clocks (i.e., PhenoAge, GrimAge, DunedinPoAm, DunedinPACE and mTL), while for fragility fractures it was seen for all clocks except DunedinPACE and mTL. Hence, PhenoAge, GrimAge, and DunedinPoAm appeared relevant for both phenotypes in this combined analysis.

#### 3.1.2 Twin Pair Level Analyses

**FIGURE 3:**
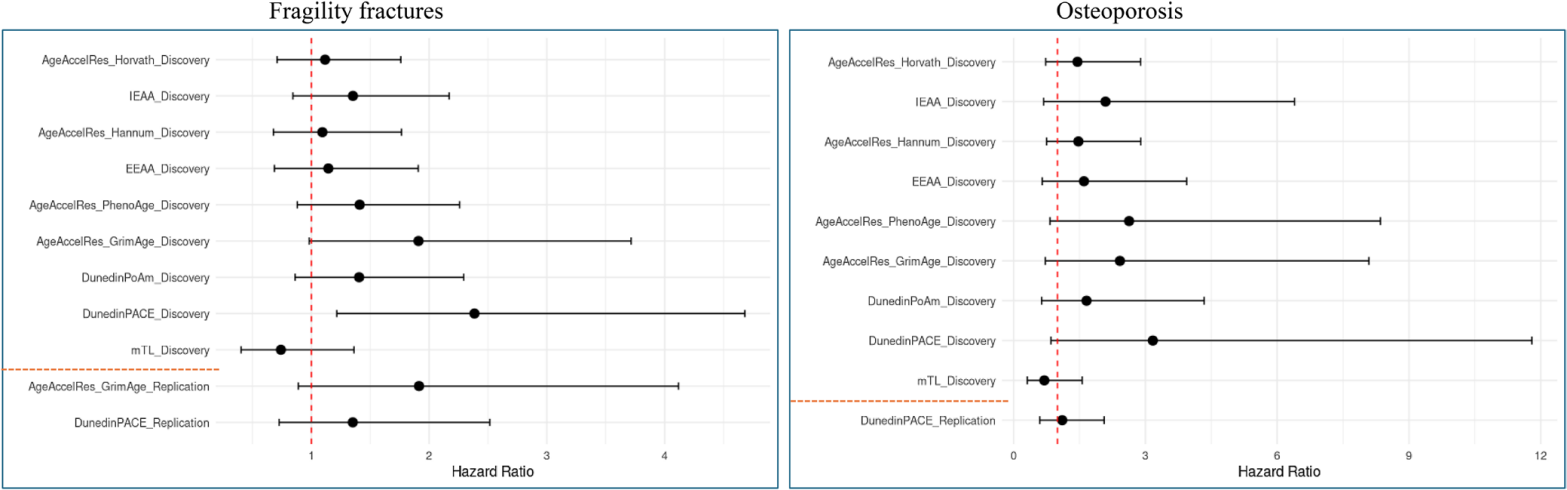
Association analysis at the twin pair level of fragility fracture and osteoporosis diagnoses after blood sampling. The red dotted line indicates a hazard ratio of 1, while the orange dotted line separates the Discovery cohort and the Replication cohort. For the Replication cohort, the epigenetic clocks with *p* values < 0.1 in the Discovery Cohort were aimed replicated.

As seen in Figure 3 and File S6, intra‐twin‐pair analysis in the Discovery cohort revealed the same directions of effects for all clocks, as had been seen in the individual‐level analysis (Figure 2); however, only DunedinPACE was significant for fragility fractures (*p* = 0.011) and borderline significant for osteoporosis (*p* = 0.090), while GrimAge was borderline significant for fragility fractures (*p* = 0.057). For both phenotypes, DunedinPACE displayed the largest effect sizes (HR = 2.39 for fragility fractures and HR = 3.17 for osteoporosis) (File S6). Investigating these associations in the Replication cohort (Figure 3, bottom) revealed the same direction of effect, although non‐significant, yet with GrimAge being borderline significant for fragility fractures (*p* = 0.095). The consistent pattern in effect sizes seen in the Discovery cohort indicates a decreased risk of obtaining a diagnosis for a co‐twin with higher mTL compared to the co‐twin with lower mTL, and for the remaining clocks an increased risk of obtaining a diagnosis for a co‐twin with higher epigenetic age compared to the co‐twin with lower epigenetic age. Hence, these directions of effect are in correspondence with our a priori hypothesis of increased biological age in individuals of impaired bone health.

For osteoporosis, the Replication cohort reflected a similar pattern for all clocks, except EEAA, while for fragility fractures it was the second and third generation clocks, especially GrimAge, DunedinPoAm, and DunedinPACE, which showed the same pattern (File S6). A combined analysis of both cohorts (File S6) moved the associations away from the null for osteoporosis for all clocks except EEAA, DunedinPACE, and mTL, while for fragility fractures a movement away from the null was seen first of all for GrimAge and DunedinPoAm. Hence, GrimAge and DunedinPoAm appear relevant for both phenotypes also in the twin pair analysis when combining both cohorts.

#### 3.1.3 Sex Stratified Analyses

As the progression of diseases of the bone potentially can be different between males and females, sex stratified analysis and a test for interaction were performed (Files S7 and S8). These analyses did not reveal statistically significant differences, that is, all *p*‐interaction values were above 0.05. However, due to only four male twin pairs with osteoporosis, the test for interaction could not be performed for osteoporosis for the twin pair model (File S8).

#### 3.1.4 Exclusion of Individuals Below 39 Years of Age

Lastly, as the associations regarding diseases of the bone might be different for premenopausal and postmenopausal women, and as the Replication cohort held two age groups (30–38, and 57–76 years of age), a sensitivity analysis excluding the youngest individuals was performed; a similar pattern in the direction of effects was observed, as compared to analyzing all individuals of the Replication cohort (Files S9 and S10).

### 3.2 Association Analyses of Bone Phenotypes and Biological Aging

#### 3.2.1 Bone Mass

**FIGURE 4:**
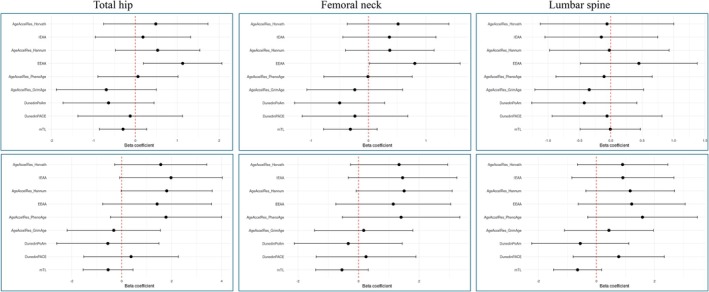
Association analysis of bone mineral density and the epigenetic clocks in the LifeSpan cohort. The red dotted line indicates a β coefficient of 0. The top panels are analysis at the individual level, while the bottom panels are analysis at the twin pair level.

For the DXA values, the epigenetic clocks did not show a consistent pattern in the directions of effects in the individual level analysis (Figure 4, top panels, and File S11), compared to that observed for the diagnoses (Figure 2). The EEAA clock did, however, show a significant association to total hip and femoral neck, with coefficients above 0, reflecting increased BMD per unit increase in standardized epigenetic age, a direction of effect that was also seen for EEAA in the twin pair analysis, although non‐significant (Figure 4, bottom panels). Positive beta coefficients were also seen with borderline significance in the twin pair analysis for the Horvath, IEAA, and Hannum clocks for total hip (*p* = 0.052–0.096), for the Hannum clock for femoral neck (*p* = 0.063), and for the PhenoAge clock for lumbar spine (*p* = 0.099) (Figure 4). This positive direction of effect is the opposite of our a priori hypothesis of worse bone health for individuals with increased epigenetic age.

**FIGURE 5:**
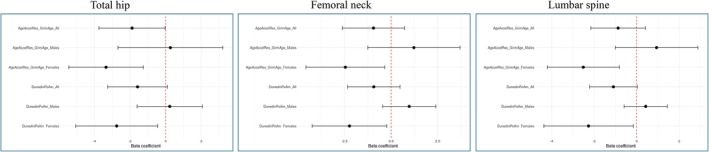
Test for interaction of sex on the association of the epigenetic clocks and DXA values in the oldest part of the LifeSpan cohort. The red dotted line indicates a β coefficient of 0. Only the GrimAge and the DunedinPoAm clock are displayed, as only these clocks showed a consistent interaction (*p* interaction: 0.009–0.053).

To investigate whether these findings could be due to sex or age differences, a number of stratifications and tests for interactions were performed. Firstly, a sex stratified analysis and test for interaction (File S12) revealed only the GrimAge clock to display a *p*‐interaction value below 0.05 (*p* = 0.037) and only in the individual‐level analysis of lumbar spine: Coef. = 0.732, *p* = 0.151 (males) and Coef. = −1.60, *p* = 0.017 (females), suggesting that in females decreased BMD is associated with increased epigenetic age, while the opposite is the case for males. Secondly, stratification and test for interaction for the younger age group (age 30–38 years) vs. the older age group (57–76) (File S13) showed none of the clocks to display a *p*‐interaction value below 0.05 in the individual‐level analysis, while two clocks did show a significant interaction in the twin‐level analysis of total hip: GrimAge (*p* = 0.043) and DunedinPoAm (*p* = 0.027), indicating increased BMD with increased epigenetic age in the younger age group (GrimAge: coef. = 2.08, *p* = 0.073, DunedinPoAm: coef. = 2.33, *p* = 0.065) and decreased BMD with increasing epigenetic age in the older age group (GrimAge: coef. = −1.83, *p* = 0.20, DunedinPoAm: coef. = −2.35, *p* = 0.133). Lastly, testing for interaction regarding sex separately within the younger or the older age groups revealed only one *p*‐interaction value below 0.05 for the youngest age group (File S14): the DunedinPACE clock in the twin pair analysis of lumbar spine (*p* = 0.043): coef. = −1.57, *p* = 0.22 (males) and coef. = 2.89, *p* = 0.16 (females), indicating decreased BMD with increasing epigenetic age in males and the opposite in females. In the oldest age group (Figure 5 and File S15), GrimAge and DunedinPoAm revealed a more consistent pattern in the *p*‐interaction values as compared to the interactions described above; these were found to be below 0.05 in the individual‐level analysis of all three BMD phenotypes with females displaying negative effect sizes and males displaying positive effect sizes. Taken together, these interaction analyses could overall suggest that the association between epigenetic age and bone mass vary both by age and sex, with the most significant negative associations observed in the females of the older age group, supporting an association in correspondence with our a priori hypothesis of increased biological age in older females with impaired bone health.

#### 3.2.2 Bone Turnover Markers

**FIGURE 6:**
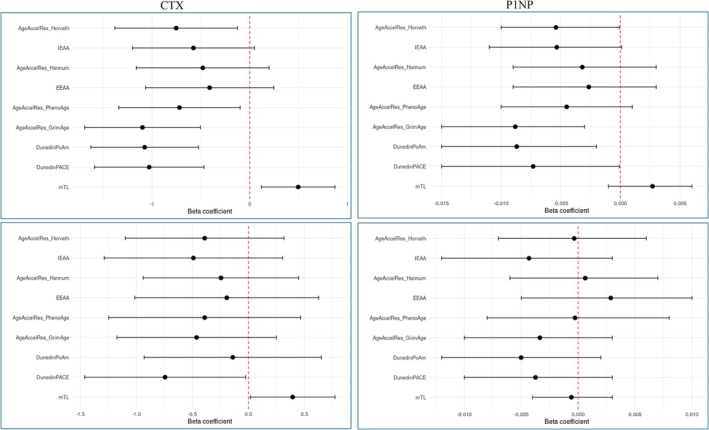
Association analysis of bone turn over markers and the epigenetic clocks in the LifeSpan cohort. The red dotted line indicates a β coefficient of 0. The top panels are analysis at the individual level, while the bottom panels are analysis at the twin pair level.

Finally, a consistent pattern in effect sizes was detected in the individual‐based analyses of the two bone turnover markers (see Figure 6, top panels, and File S16), with mTL displaying coefficients above 0, indicating an increased level of CTX and P1NP per unit increase in mTL. The remaining clocks displayed the opposite direction, that is, decreased level of CTX and P1NP per unit increase in standardized epigenetic age, although not all clocks were statistically significant. The twin pair level analysis overall revealed the same direction of effect, but only mTL and DunedinPACE were statistically significant for CTX (Figure 6, bottom panels, and File S16). For P1NP, this pattern in the direction of effect is in correspondence with our a priori hypothesis of increased biological age in individuals of worse bone health, as P1NP is a marker for bone formation, while for CTX it is against our a priori hypothesis, as CTX is a marker for bone resorption.

Hence, for consistency with the analyses of DXA, stratification and test for interaction regarding either sex or the younger versus the older age group were performed in order to examine whether this finding was due to sex or age effects (Files S17 and S18). Only one *p*‐interaction value below 0.05 was found for sex: IEAA (*p* = 0.029) in the twin pair level analysis of CTX: coef. = −1.380, *p* = 0.036 (males), and coef. = 0.365 *p* = 0.433 (females), indicating decreased level of CTX by increasing epigenetic age in males and increased level of CTX by increasing epigenetic age in females, the latter being in correspondence with our a priori hypothesis. No *p*‐interaction values below 0.05 were seen regarding the young vs. the older age group (File S18). Finally, stratification by sex within the younger or the older group (Files S19 and S20) did not reveal any *p*‐interaction values below 0.05 for the older group, while in the younger group in individual level analysis of CTX the DunedinPACE clock displayed a *p*‐interaction value of 0.004 with coef. = −0.052, *p* = 0.9185 (males) and coef. = −3.11, *p* = 0.0004, simply indicating a larger negative effect size in females. Taken together, these interaction analyses could overall suggest sex and age effects; however, due to few clocks showing significance, the pattern appears somehow inconsistent.

## 4 Discussion

Osteoporosis is considered a disease of bone age acceleration, a process believed to be affected by epigenetic mechanisms. Therefore, a higher epigenetic age is expected in patients with osteoporosis, or individuals experiencing a fragility fracture, compared to healthy individuals. However, studies examining this hypothesis are surprisingly rare and usually suffer from small sample size [32 osteoporosis patients, and 16 controls (Fernandez‐Rebollo et al. 2018)], or 72 adults for investigating BMD (Shiau et al. 2024), or were performed in children (Simpkin et al. 2017). Here, we examined the association of epigenetic age to osteoporosis and fragility fracture diagnoses in 1087 twins, as well as to bone mass measurements and levels of bone turnover markers in 288 twins.

The findings for the diagnoses reflected our a priori hypothesis, that is, HRs above 1 indicating an increased risk of diagnosis for age‐accelerated individuals, and a HR below 1 for mTL reflecting a decreased risk of diagnosis for individuals with longer telomeres; findings that overall reflect a higher biological aging among individuals of worse bone health. This consistent pattern was also seen in the Replication cohort, despite their younger age (Discovery cohort: 73–91 years, mean = 81 years, Replication cohort: 30–79 years, mean = 60 years). Such a consistent pattern is likely not a chance finding; however, additional studies are needed to corroborate our findings.

We observed that DunedinPoAm and DuninPACE showed consistent findings across diagnosis groups. Both clocks were developed by analyzing DNA methylation data and longitudinal biochemical data from the Dunedin Study participants (Belsky et al. 2020, 2022). In the present study, they seem to capture bone age acceleration more precisely than the first‐generation clocks based on cross‐sectional data, for example, Horvath and Hannum, or the aging clock, that is, PhenoAge. In addition to the Dunin clocks, GrimAge also showed consistency, especially for fragility fractures; perhaps not surprisingly considering that GrimAge was generated (Lu, Seeboth, et al. 2019) against age‐related biomarkers (e.g., TIMP‐1, Cystatin C, GDF‐1), smoking pack‐years, and time‐to‐death, likely reflecting a higher accelerated bone fragility in the individuals who experience a bone fracture as compared to individuals who have osteoporosis. Nevertheless, in general, the analysis of osteoporosis showed the smallest *p* values, that is, being the furthest away from the a priori null hypothesis, reflecting that the clocks capture age‐related changes with regard to osteoporosis more precisely than fragility bone fractures.

We included twin pair analysis in our study in order to reduce the genetic and environmental confounding; and the findings of the twin pair level analyses supported the findings of the individual level analyses. This observation highlights the strength of including twin pairs in molecular epidemiological studies as compared to including singletons only and corroborates our previous studies of other traits (e.g., Mak et al. 2024; Soerensen et al. 2022). The twin studies supported our a priori hypothesis and supported our individual‐based analysi; especially GrimAge, DunedinPoAm, and DunedinPACE appear more sensitive in identifying individuals with impaired bone health.

Interestingly, for BMD the pattern was less consistent with some clocks displaying positive beta coefficients, indicating that age‐accelerated individuals have higher BMD. This is against our a priori hypothesis. However, one plausible explanation is that the LifeSpan cohort consists of both premenopausal and postmenopausal women. Stratification and interaction analysis of age and sex‐specific effects supported this notion as the DunedinPoAm and GrimAge clocks showed negative beta coefficients, corresponding to an increased age acceleration with decreased BMD in the older postmenopausal women of the cohort. Hence, the relation between bone mass and the epigenetic clocks appears both age‐ and sex‐dependent. Regarding P1NP, a bone formation marker, and CTX, a bone resorption marker, we observed that higher epigenetic age is associated with decreased bone marker levels. This contradicts our a priori hypothesis with respect to CTX. However, it is important to keep in mind that bone mass is dependent on a balance between bone formation and resorption, and the direction seen for P1NP and CTX might simply reflect a low bone turnover in individuals with a high biological age. Finally, our data on the bone phenotype are cross‐sectional, and to fully examine this relationship, longitudinal data over several years are needed.

Our study has a number of strengths. First, it is the largest study to date, and the first to apply twin samples, systematically examining the association of epigenetic clocks with bone aging. Second, we use nationwide register data on diagnoses, leading to a less biased investigation compared to self‐report. It furthermore enables the investigation of incident cases. On the other hand, our study has a number of limitations. First, the epigenetic clocks were based on blood samples, which means that the clocks reflect the overall biology of an individual, and not the biology specific to bone tissue. However, molecular biomarkers measured in blood are clinically relevant due to the ease of obtaining a blood sample compared to obtaining a bone tissue sample, which requires an invasive bone tissue biopsy. Secondly, even though the cases of the present study were defined as obtaining a diagnosis after blood sampling, yet not having a diagnosis before blood sampling, we cannot exclude that pathological changes were already present at the time of blood sampling, as osteoporosis develops over many years. This is especially relevant for the present study due to the changes in the definitions of bone diagnoses over time, that is, the ICD‐10 code definitions used in the present study are not easily transferred to ICD‐8 codes back in time, that is, back to the beginning of the Danish National Patient Registry in 1977. Furthermore, the diagnoses in the registry are provided by medical doctors in the Danish hospital system; consequently, the diagnoses are population‐based and nationwide. Due to the nature of the register data, we do however not have detailed information from patient records; consequently, we do not have detailed information on whether a fracture was minor or major or detailed information on its cause. However, previous studies have demonstrated a high validity of osteoporotic fracture diagnoses in the registry data, including a high correspondence to patient record data from hospitals (Clausen et al. 2024). A third limitation is that data on BMD was only available for the youngest twins, but not for the middle‐aged and older twins, as the MADT and LSADT cohorts were large population‐based cohorts with an overall focus on aging, that is, they did not include DXA scanning. Hence, it was not possible to thoroughly investigate osteoporosis based on BMD measurements in the relevant age group. Lastly, in the present study we had an a priori hypothesis about the direction of effect, that is, increased epigenetic age for individuals with impaired bone health, and expected beta coefficients above 0 and hazard ratios above 1 in the analyses of the clocks (and the opposite for mTL), which for the diagnoses to a large extent was what we observed. Despite our a priori hypothesis, we did calculate 2‐sided *p* values; yet we did not perform correction for multiple testing as the epigenetic clocks are not independent, that is, they all contain different measures of biological age.

In conclusion, the present study supports an accelerated aging phenotype, as reflected in the epigenetic clocks, for individuals with fractures or osteoporosis phenotypes. Although most of the directions of effect observed are consistent and in correspondence with our a priori hypothesis, additional studies are needed for verification. This is especially important considering that such studies on bone phenotypes are still surprisingly rare within this field of research.

## Author Contributions

M.S. performed all data analyses, wrote the original draft, and did project administration. All authors conducted conception and design of the study, interpretation of the results, review and editing of the draft, and have read and approved the final version of the manuscript. K.C. performed data acquisition.

## Ethics Statement

Informed consent was obtained from all participants, and the surveys were approved by the Regional Scientific Ethical Committees for Southern Denmark (S‐VF‐19980072, S‐VF‐20040241, and S‐20090033), and conducted in accordance with the Helsinki II declaration.

## Conflicts of Interest

The authors declare no conflicts of interest.

## Supporting information


**Data S1:** Detailed description of materials and methods.

**File S1:** Descriptions of the epigenetic clocks investigated in the present study.
**File S2:** International Classification of Diseases 10 codes from the Danish National Patient Registry.
**File S3:** Characteristics of the LifeSpan cohort.
**File S4:** Two‐way scatter plots of the epigenetic clocks and chronological age investigated in the present study.
**File S5:** Association analysis of diagnoses after blood sampling and the epigenetic clocks in the Discovery Cohort, the Replication Cohort and combined sample (individual‐level‐analysis).
**File S6:** Association analysis of diagnoses after blood sampling and the epigenetic clocks in the Discovery Cohort, the Replication Cohort, and the combined sample (twin‐pair‐level‐analysis).
**File S7:** Association analysis of diagnoses after blood sampling and the epigenetic clocks in the Discovery Cohort, stratified by sex (individual‐level‐analysis).
**File S8:** Association analysis of diagnoses after blood sampling and the epigenetic clocks in the Discovery Cohort, stratified by sex (twin‐pair‐level‐analysis).
**File S9:** Association analysis of diagnoses after blood sampling and the epigenetic clocks in the Replication Cohort, analysis of all individuals and excluding the youngest individuals of the LifeSpan cohort (individual‐level analysis).
**File S10:** Association analysis of diagnoses after blood sampling and the epigenetic clocks in the Replication Cohort, analysis of all individuals and excluding the youngest individuals of the LifeSpan cohort (twin‐pair‐level analysis).
**File S11:** Association analysis of DXA scan values and the epigenetic clocks in the LifeSpan Cohort.
**File S12:** Association analysis of DXA scan values and the epigenetic clocks in the LifeSpan Cohort, stratified by sex.
**File S13:** Association analysis of DXA scan values and the epigenetic clocks in the LifeSpan Cohort, stratified by youngest (age 30–38) and oldest (57–76) age group.
**File S14:** Association analysis of DXA scan values and the epigenetic clocks in the youngest part of the LifeSpan Cohort, stratified by sex.
**File S15:** Association analysis of DXA scan values and the epigenetic clocks in the oldest part of the LifeSpan Cohort, stratified by sex.
**File S16:** Association analysis of bone biomarkers and the epigenetic clocks in the LifeSpan Cohort.
**File S17:** Association analysis of bone biomarkers and the epigenetic clocks in the LifeSpan Cohort, stratified by sex.
**File S18:** Association analysis of bone biomarkers and the epigenetic clocks in the LifeSpan Cohort, stratified by youngest (age 30–38) and oldest (57–76) age group.
**File S19:** Association analysis of bone biomarkers and the epigenetic clocks the youngest part of the LifeSpan Cohort, stratified by sex.
**File S20:** Association analysis of bone biomarkers and the epigenetic clocks the oldest part of the LifeSpan Cohort, stratified by sex.

## Data Availability

According to Danish and EU legislation, the transfer and sharing of individual‐level data requires prior approval from the Danish Data Protection Agency and requires that data sharing requests be dealt with on a case‐by‐case basis. Therefore, the data from the present study cannot be deposited in a public database. However, we welcome any inquiries regarding collaboration and individual requests for data sharing.
