# Supplementary material for: Epigenetic Clocks as Biomarkers for Bone Aging: Evidence From a Twin Study
Source: Aging Cell. 2025 Sep 14;24(10):e70204. doi: 10.1111/acel.70204 (PMC12507416; doi:10.1111/acel.70204)
Supplement: Supplementary file 1 — Data S1: Detailed description of materials and methods. [file ACEL-24-e70204-s001.docx]

**Detailed Description of Materials and Methods**

**Study Population**

The study population included 1,087 twins drawn from three population-based, nationwide surveys from the Danish Twin Registry (Pedersen et al., 2019): The Longitudinal Study of Aging Danish Twins (LSADT), The Middle Age Danish Twin study (MADT), and the Birthweight-Discordant Study (hereon called LifeSpan). LSADT was started in 1995 as a cohort-sequential study of Danish twins above 70 years of age, where surviving twins were followed up every second year until 2005 (McGue and Christensen 2002). In 2007 all twin pairs, where both twins were still alive, were invited to participate. The MADT was initiated in 1998, and comprised twins randomly selected from birth cohorts spanning 1931–1952. In 2008–2011 a follow-up study was conducted of all eligible twin pairs originally enrolled (Pedersen et al., 2019). The LifeSpan cohort was performed in 2009–2011, and included twin pairs from the registry, who had a median intra-pair difference in birth weight of 0.5 kg. (Frost et al., 2012). The LifeSpan twins have previously been included in an epigenome-wide association study of birth-weight discordance by Tan et al. (2014), who found no significant CpGs. The present study includes twins from the three cohorts for whom genome-wide epigenetic, and register data are available: 224 twins from the LSADT 1997 wave, 86 twins from the LSADT 2007 wave, 489 twins from the MADT 2008-2011 wave, and 288 twins from the Lifespan cohort. The LSADT twins composed the Discovery Sample (N=310), while the MADT and LifeSpan cohorts composed the Replication sample (N=777). Zygosity had been determined either by microsatellite markers, or genome-wide genetic data (LifeSpan, and LSADT), or by four questions regarding physical similarity (MADT). The latter has been reported to correctly classify more than 95% of the twin pairs (Christiansen et al., 2003).

For all cohorts, data were collected via comprehensive interview-based questionnaires, and examinations, as well as blood sampling. In addition, comprehensive biochemical testing, as well as dual-energy X-ray absorptiometry (DXA) scanning were performed in the LifeSpan cohort. Data on disease diagnoses, and survival status were obtained through linkage to nationwide health registries. Informed consent was obtained from all participants, and the surveys were approved by the Regional Scientific Ethical Committees for Southern Denmark (S-VF-19980072, S-VF-20040241, and S-20090033), and conducted in accordance with the Helsinki II declaration.

**Diagnoses of Bone Fractures and Osteoporosis from the Danish National Patient Registry**

Disease diagnoses were gathered by extracting International Classification of Diseases (ICD) codes from the Danish National Patient Registry (DNPR), which holds all hospital discharges, and outpatient visits from all Danish hospitals since 1977 (see https://sundhedsdatastyrelsen.dk/da/english). The disease diagnoses in DNPR are classified according to ICD codes, with ICD8, and ICD10 codes, the latter since 1994. ICD-9 codes were never used in Danish registries. In the present study ICD10 codes were available until the 23^rd^ of November 2022. Both primary and secondary diagnoses were used, and were grouped into two disease groups:

(1) Fragility fractures: M484, M485, M495, M800-M805, M808, M809, S120-S122, S127-S129, S220, S221, S320-S325, S327, S328, S422-S424, S520-S529, S720-S722, and T08.

(2) Osteoporosis: M810-816, M818-821, M828, M858, and M859.

The details on the specific codes can be found in Supplementary Information, Sheet 2.

Within each of these two disease groups, the first occurring diagnose date was defined per person, and the individuals were divided into prevalent, and incident cases, i.e., whether the first occurring diagnose date was before (prevalent), or after (incident) blood sample collection. As the focus of the present study was on incident disease, only the incident cases were included for statistical analysis, i.e., individuals holding minimum one diagnoses after blood sampling (blood sampling took place in 1997 or 2007 (LSADT) and 2008-2011 (MADT/LifeSpan)), yet no diagnoses before blood sampling (diagnose coverage back to 1994) within the disease group of interest, were included. The prevalent cases were excluded from analysis. Similarly, within each disease group, the twin pairs discordant for incident diagnose status were found; these twin pairs are the twin pairs where one co-twin received a diagnosis after blood sampling, while the co-twin remained disease free, as well as twin pairs where both twins received a diagnosis, yet at different time points after blood sampling. Finally, with the purpose to investigate time to diagnosis after blood sampling, mortality status was obtained from the Danish Central Person Register (see https://sundhedsdatastyrelsen.dk/da/english), assessed also on the 23^rd^ of November 2022; 304 out of the 310 individuals of the Discovery Sample had died, while the number was 125 of out the 777 members of the Replication samples. Two members of the Replication cohort had emigrated and were hence censored at this date in the statistical analysis.

**Biological data**

**DNA Methylation Data**

A detailed description of the laboratory methods, and quality control of the Infinium HumanMethylation450K BeadChip (Illumina, San Diego, CA, United States) methylation data can be found in Soerensen et al. (2019). In short, 500 ng DNA per sample was bisulfite converted using the EZ Methylation Gold kit (Zymo Research, Orange County, CA, United States) prior array analysis using standard procedures. Quality control was conducted using the MethylAid (van Iterson et al. 2014), and Minfi (Aryee et al., 2014) R packages. Sample exclusion criteria were: (a) <95% of probes with a detection p-value < 0.01, (b) samples failing the internal quality control probes of the MethylAid, or (c) samples failing verification of sex by multidimensional scaling of the X chromosome probe values. For all individuals of the study population the sex assigned at birth (as defined by the registries) was confirmed in the latter analysis. Probe exclusion criteria were: (a) detection p-value > 0.01, (b) a raw intensity value of zero, (c) low bead count (<3 beads), (d) cross-reactive probes (as defined by Chen et al., (2013)), or (e) measurement success rate < 95%. Normalization was performed with Functional normalization (Fortin et al., 2014). DNA methylation was measured at four separate occasions, with twin pairs run on the same array. Quality control was performed individually for each of the four datasets.

**DNA Methylation Derived Estimates of Biological Age, Pace of Ageing and Leukocyte Telomere Length**

We calculated six different measures of epigenetic age measured in the units of years (see Supplementary Information, Sheet 1 for description): Horvath (Horvath, 2013), Hannum (Hannum et al., 2013), PhenoAge (Levine et al., 2018), GrimAge (Lu et al., 2019a), intrinsic epigenetic age acceleration (IEAA), and extrinsic epigenetic age acceleration (EEAA) (Chen et al., 2016). While the first-generation clocks Horvath, and Hannum, and their derivatives IEAA, and EEAA, were generated as predictors of chronological age by using chronological age as outcome in prediction modelling, the second-generation clocks PhenoAge, and GrimAge were developed with the aim to reflect aging, and time-to-death, respectively. For PhenoAge a Phenotypic age variable was obtained based on clinical data, and measurements of several biochemical markers, and subsequently used in the predictor modeling (Levine et al., 2018). GrimAge is technically speaking a mortality risk estimator: DNA methylation-based surrogates for plasma proteins, and smoking pack years were generated, and subsequently regressed against time-to-death. Associated markers were then used to generate a composite measure of time-to-death, which was finally transformed into an age in units of years (Lu et al., 2019a). Of these six estimators, Horvath, and IEAA are considered intrinsic, i.e., they reflect cell-type independent effects of aging on DNA methylation, while Hannum, EEAA, PhenoAge, and GrimAge are considered extrinsic measures, i.e., they also reflect age-related changes to the immune-cell composition of the peripheral blood (Chen et al., 2016). Specifically, the IEAA is the residuals achieved by regressing chronological age, and DNA methylation-derived estimates of cell counts, i.e., plasma B cells (variable PlasmaBlast), CD8^+^CD28^-^CD45RA T cells (exhausted CD8^+^ T cells) (variable CD8pCD28nCD45Ran), naïve CD8+ T cells (variable CD8_naive), CD4^+^ T cells (variable CD4T), natural killer cells (variable NK), monocytes (variable Mono), and granulocytes (variable Gran) onto the Horvath age estimator (Horvath, 2013, Chen et al., 2016). The EEAA is obtained by regressing chronological age on to the BioAge4HAStatic variable, which is a modified version of the Hannum age estimator achieved as a weighted average of the Hannum age estimator, and three estimated measures of age-associated blood cells, i.e., naïve (CD45RA^+^CCR7^+^) cytotoxic T cells, exhausted (CD28^-^CD45RA^-^) cytotoxic T cells, and plasma B cells (Chen et al., 2016).

Moreover, in addition to the above-mentioned six estimators defined in units of years, we estimated three additional estimators not measured in units of years: two measures of the pace of biological aging (DunedinPoAm (Belsky et al., 2020) and DunedinPACE (Belsky et al., 2022)), and one of leucocyte telomere length (mTL (Lu et al., 2019b)). DunedinPoAm was the first clock to include longitudinal data, precisely measurements of 18 biomarkers measured 3 times over a 12-year period (age 26 to 38), generating the so-called pace-of-aging, i.e., a rate of change in these biomarkers. Subsequently the pace-of-aging was used in prediction modelling against DNA methylation data, deriving DunedinPoAm meant to reflect a person’s pace of biological aging (Belsky et al., 2020). Subsequently, the Dunedin clock was refined to include 19 biomarkers measured four times over a broader age span (26 to 46 years) and only using DNA methylation sites with acceptable test-retest reliability in both 450K and EPIC array data (Belsky et al. 2022). mTL was obtained using leukocyte telomere length (LTL) measurements, either based on quantitative real-time polymerase chain reaction, or Southern blotting procedures, as outcome in prediction modelling. As such mTL is a surrogate for LTL and is measured in units of kilo bases (kb) (Lu et al., 2019b).

In the present study the Horvath, Hannum, PhenoAge, GrimAge, and mTL estimators were computed as principal component (PC) clocks using the method published by Higgins-Chen et al. (2022), with the aim to increase the accuracy of the clocks. Missing values were imputed using the dataset by Hannum et al. (2013) (https://www.ncbi.nlm.nih.gov/geo/query/acc.cgi?acc=GSE40279), as suggested by Higgins-Chen et al. (2022). As DunedinPoAm38, DunedinPACE and EEAA are not included in the PC algorithm (Higgins-Chen et al., 2022), DunedinPoAm38 and DunedinPACE were obtained as described in the original publications (Belsky et al., 2020 and Belsky et al. 2022), while EEAA was acquired using scripts kindly provided by Steve Horvath, and Ake Lu (University of California). The methylation-based cell counts used for calculation of IEAA, and EEAA were obtained via the online DNA methylation age calculator (https://dnamage.genetics.ucla.edu/home).

To ensure statistical analyses in a consistent manner, we defined epigenetic age acceleration (AgeAccel) as the residuals obtained from regressing the estimators on chronological age for the clock measured in units of years. As seen in Supplementary Information, Sheet4; as expected the epigenetic clocks in general correlated to the chronological age, while the AgeAccels did not. Before statistical analysis, all clocks were standardized to a mean of 0 and a standard deviation of 1. This was done separately for the Discovery cohort, and the Replication cohort for analysis of diagnoses, and separately for the LifeSpan cohort for analysis of bone mineral density (BMD) and bone turnover markers.

**Measurements of Bone Biology in the LifeSpan Cohort**

The twins of the LifeSpan cohort (see Supplementary Information, Sheet 3 for descriptives) underwent blood sampling, survey questionnaire, and measurements similar to the MADT cohort, yet additionally underwent whole body DXA scanning. Furthermore, their blood samples were analyzed for several biochemical markers of metabolism, and bone biology (Frost et al., 2012, Frost et al., 2013a, Frost et al., 2013b). In the present study DXA-based measurements (g/m^2^) of bone mineral density of the total hip, the femoral neck of the hip, and the lumbar spine were analyzed for their association to the epigenetic clocks. So were blood-based values (μg/L) for the bone turnover markers cross-linked C-telopeptide (CTX), and amino-terminal propeptide of type I procollagen (P1NP).

**Statistical Analyses**

The study population was analyzed both at the individual level, i.e., as singletons, and at the twin pair level, the latter with the purpose of investigating intra-pair differences, i.e., to make use of the discordant twin pair design. All analyses were performed using STATA17 (Stata Corporation, College Station, TX, USA), and all reported P values are two-sided, and not adjusted for multiple testing. We defined statistical significance as P < 0.05.

First, we explored the association between the epigenetic clocks, and time to diagnoses at the individual level by performing Cox proportional hazards regression with age as the timescale (delayed entry at blood collection) and adjusting for sex by using sex-specific baseline hazards. To account for dependency between twins in each twin pair, we used the robust estimator of variance, assuming independence between pairs (cluster function in STATA). Furthermore, based on the FRAX score evaluating the risk of fracture (de Laet et al., 2005) we performed two additional analyses, (1) a model, which in addition to age, and sex, was adjusted for current smoking status (yes/no), and drinking habits (< or ≥ 3 units per day), and (2) a model further adjusting for height (cm), and weight (kg), as well as a Charlson co-morbidity index (Christensen et al., 2011). The Charlson co-morbidity index was estimated using all primary ICD8 and ICD10 codes, as defined by the index, with a diagnose date before blood sampling. The disease codes were obtained from the DNPR. Hence, the index reflects the disease load, which an individual has encountered at time of blood sampling. Smoking and alcohol habits were based on self-report, while height and weight were measured in the LifeSpan and MADT cohorts, yet self-reported in the LSADT cohort. Moreover, for analysis at the twin pair level, i.e., for investigation of the intra-pair differences, a stratified Cox regression model (stratifying by twin pair ID) was used adjusting for the same co-variates as described above. By investigating intra-pair differences, this twin pair level analysis adjusts for the environmental and genetic factors shared by the twins in a pair.

Finally, in the LifeSpan cohort linear regression was performed to analyze the association between bone phenotypes, and the epigenetic clocks as outcome. The association was, similarly to the Cox regression analyses, adjusted for age, and sex, as well as further adjusting for smoking, and alcohol habits, and furthermore for height, and weight, and the Charlson co-morbidity index. To account for dependency between twins in a pair we used the Hubert-White-Sandwich (robust) estimator of variance, assuming independence between twin pairs (cluster function in STATA). In the twin pair level analysis, intrapair differences were investigated by fitting a fixed-effects model with a within twin pair regression estimator (xtreg command with fe option in STATA). The epigenetic clocks investigated with respect to the bone phenotypes were calculated separately for the LifeSpan cohort.

All results of the present study are listed as tables, as well as forest plots, in the Supplementary Information, Sheets 5-20. Forest plots were generated in R (version 4.4.1) using the ggplots2 library.

**References**

Aryee MJ, Jaffe AE, Corrada-Bravo H et al. Minfi: A flexible and comprehensive Bioconductor package for the analysis of Infinium DNA methylation microarrays. *Bioinformatics* 2014;30(10):1363-1369. doi:10.1093/bioinformatics/btu049.

Belsky DW, Caspi A, Arseneault L et al. Quantification of the pace of biological aging in humans through a blood test, the DunedinPoAm DNA methylation algorithm. *Elife* 2020;9:e54870, doi: 10.7554/eLife.54870.

Belsky DW, Caspi A, Corcoran DL, et al. DunedinPACE, a DNA methylation biomarker of the pace

of aging. *Elife* 2022; 14:11:e73420, doi: 10.7554/eLife.73420.

Chen YA, Lemire M, Choufani S et al. Discovery of cross-reactive probes and polymorphic CpGs in the Illumina Infinium HumanMethylation450 microarray. *Epigenetics* 2013;8(2):203-209. doi:10.4161/epi.23470.

Chen BH, Marioni RE, Colicino E et al. DNA methylation-based measures of biological age: Meta-analysis predicting time to death *Aging (Albany NY)* 2016;8(9):1844-1865. doi:10.18632/aging.101020.

Christensen S, Johansen MB, Christiansen CF, Jensen R, Lemeshow S. Comparison of Charlson comorbidity index with SAPS and APACHE scores for prediction of mortality following intensive care. *Clin Epidemiol* 2011;3:203-11. doi: 10.2147/CLEP.S20247.

Christiansen L, Frederiksen H, Schousboe K, et al. Age- and Sex-differences in the Validity of Questionnaire-based Zygosity in Twins. *Twin Res* 2003, 6(4): 275-278. doi: 10.1375/136905203322296610.

De Laet C, Oden A, Johnell O, Jonsson B, Kanis JA. The impact of the use of multiple risk factors on case finding strategies: a mathematical framework. *Osteoporos Int* 2005; 16: 313-318. doi: 10.1007/s00198-004-1689-z.

Fortin J, Labbe A, Lemire M et al. Functional normalization of 450k methylation array data improves replication in large cancer studies. *Genome Biol* 2014;15(12):503. doi: 10.1186/s13059-014-0503-2.

Frost, M, Petersen I, Brixen K et al. Adult glucose metabolism in extremely birthweight-discordant monozygotic twins. *Diabetologia* 2012, 55(12): 3204-12. doi: 10.1007/s00125-012-2695-x.

Frost M, Petersen I, Andersen TL et al. Birth weight and adult bone metabolism are unrelated: results from birth weight-discordant monozygotic twins. *J Bone Miner Res.* 2013;28(12):2561-9. doi: 10.1002/jbmr.1995.

Frost M, Petersen I, Hegedüs L, Christiansen L, Brix T, Christensen K. Regulation of the pituitary-thyroid axis in adulthood is not related to birth weight: evidence from extremely birth weight-discordant monozygotic Danish twin pairs. *Thyroid* 2013;23(7):785-90. doi: 10.1089/thy.2012.0095.

Hannum G, Guinney J, Zhao L et al. Genome-wide Methylation Profiles Reveal Quantitative Views of Human Aging Rates. Mol Cell 2013;49(2):359-367. doi:10.1016/j.molcel.2012.10.016.

Higgins-Chen AT, Thrush KL, Wang Y et al. A computational solution for bolstering reliability of epigenetic clocks: Implications for clinical trials and longitudinal tracking. *Nat Aging* 2022;2(7):644-661. doi: 10.1038/s43587-022-00248-2.

Horvath S. DNA methylation age of human tissues and cell types. Genome Biol 2013;14(10):R115. doi:10.1186/gb-2013-14-10-r115.

Levine ME, Lu AT, Quach A et al. An epigenetic biomarker of aging for lifespan and healthspan. *Aging (Albany NY)* 2018;10(4):573-591. doi:10.18632/aging.101414.

Lu AT, Quach A, Wilson JG et al. DNA methylation GrimAge strongly predicts lifespan and healthspan. *Aging (Albany NY)* 2019;11(2):303-327. doi:10.18632/aging.101684.

Lu AT, Seeboth A, Tsai PC et al. DNA methylation-based estimator of telomere length. *Aging (Albany NY)* 2019;11(16):5895-5923. doi: 10.18632/aging.102173.

McGue, M. and K. Christensen. The heritability of level and rate-of-change in cognitive functioning in Danish twins aged 70 years and older. Exp Aging Res 2002, 28(4): 435-51. doi: 10.1080/03610730290080416.

Pedersen, DA, Larsen LA, Nygaard M et al. The Danish Twin Registry: An Updated Overview. *Twin Res Hum Genet* 2019, 22(6): 499-507. doi: 10.1017/thg.2019.72.

Soerensen M, Li W, Debrabant B et al. Epigenome-wide exploratory study of monozygotic twins suggests differentially methylated regions to associate with hand grip strength. *Biogerontology* 2019, 20(5):627-647. doi: 10.1007/s10522-019-09818-1.

Tan Q, Frost M, Heijmans BT et al. Epigenetic signature of birth weight discordance in adult twins. *BMC Genomics* 2014. 15(1): 1062. doi: 10.1186/1471-2164-15-1062.

Van Iterson M, Tobi EW, Slieker RC et al. MethylAid: Visual and interactive quality control of large Illumina 450k datasets. *Bioinformatics* 2014;30(23):3435-3437. doi:10.1093/bioinformatics/btu566.
